# Low Afterglow Composite Scintillator for Real-Time X-Ray Imaging

**DOI:** 10.3390/ma19020437

**Published:** 2026-01-22

**Authors:** Xiangzhou Zhang, Yeqi Liu, Nianqiao Liu, Zhaolai Chen, Yuhai Zhang, Xiao Cheng

**Affiliations:** 1 School of Materials Science and Engineering, Shandong University, Jinan 250061, China; xz_zhang@sdu.edu.cn; 2 Institute for Advanced Interdisciplinary Research (IAIR), School of Chemistry and Chemical Engineering, University of Jinan, Jinan 250022, China; chm_liuyq@ujn.edu.cn (Y.L.); ifc_zhangyh@ujn.edu.cn (Y.Z.); 3 School of Materials Science and Engineering, University of Jinan, Jinan 250100, China; mse_liunq@ujn.edu.cn; 4 State Key Laboratory of Crystal Materials, Institute of Crystal Materials, Shandong University, No. 27 Shanda South Road, Jinan 250100, China; zhaolaichen@sdu.edu.cn

**Keywords:** X-ray imaging, low afterglow, Ce^3+^/Tb^3+^ co-doped NaLuF_4_

## Abstract

Rare-earth fluoride nanocrystals have emerged as promising scintillator materials due to their excellent optical properties, environmental stability, and ease of fabrication into flexible screens. However, their practical application is often hindered by persistent afterglow, a phenomenon caused by deep trap states that capture and slowly release charge carriers after X-ray excitation, which leads to signal overlap and image artifacts in dynamic imaging scenarios. This study addresses this critical challenge by developing Ce^3+^/Tb^3+^ co-doped NaLuF_4_ nanoscintillators with suppressed afterglow. By introducing Ce^3+^ions as dopants into the Tb^3+^-activated NaLuF_4_ host, we successfully quenched the characteristic long afterglow without compromising the intrinsic radioluminescence efficiency of the Tb^3+^ centers. The optimized nanocrystals were subsequently incorporated into a poly (vinyl alcohol) matrix to fabricate transparent, high-loading composite scintillator films. The resulting films exhibit negligible afterglow, maintain high spatial resolution, and demonstrate excellent radiation stability. This work presents an effective strategy for suppressing afterglow in rare-earth fluoride scintillators through targeted ion doping, which paves the way for their application in real-time, high-quality X-ray imaging technologies such as medical diagnostics and industrial inspection.

## 1. Introduction

Rare-earth fluoride scintillators have attracted considerable attention in recent years owing to their superior optical properties and environmental stability, rendering them applicable in fields such as bio-imaging, medical diagnostics, and industrial inspection [1,2,3,4,5,6,7]. In particular, rare-earth fluoride nanocrystals feature facile preparation, which enables the fabrication of flexible scintillator screens [8,9,10]. For example, Ou et al. reported the integration of rare-earth fluoride scintillators with polydimethylsiloxane to fabricate a transparent film, achieving high-resolution 3D imaging [11]. Thus, benefiting from the high X-ray attenuation and light yield, rare-earth fluoride scintillators are suitable candidate materials for scintillator applications [12,13,14,15,16].

Although rare-earth fluorides exhibit favorable properties for X-ray imaging, they face a significant challenge in the form of afterglow [17,18,19,20,21]. Afterglow originates from deep trap states, such as Frenkel defects (F-ion vacancies and interstitials), which form within the crystal lattice upon excitation by high-energy X-rays [22]. These defects trap electrons and cause their slow release, resulting in persistent luminescence that can last from hours to days [15,23,24,25,26]. Such prolonged afterglow overlaps with subsequent imaging signals, thereby reducing the timeliness and accuracy of dynamic imaging, especially in scenarios that require rapid responses, such as medical computed tomography scans, where it may lead to image blurring or artifacts [27,28].

So far, researchers have adopted various strategies to mitigate the long afterglow of scintillating materials [14,29,30,31,32]. For instance, Zhang et al. used Cu^+^ doping to suppress the X-ray-induced afterglow in Cs_2_LiYCl_6_: Ce crystals, thereby decreasing the concentration of deep trap states and shortening the afterglow duration [31]. Similarly, rare earth doping is used to suppress the afterglow of phosphors. Tian et al. successfully synthesized a series of Sb^3+^, Bi^3+^, Sm^3+^, and Yb^3+^ doped RbI: Tl scintillators, which exhibited enhanced light yield and weakened afterglow characteristics [32]. Consequently, the co-doping strategy serves as a highly viable and effective method for substantially suppressing the afterglow frequently encountered in scintillators, and it has emerged as a crucial technique for advancing scintillator development.

In this study, well-dispersed NaLuF_4_ nanocrystals were synthesized via a solvothermal method. By introducing Ce^3+^, we successfully suppressed the X-ray-induced long afterglow typically observed in Tb^3+^-doped NaLuF_4_ systems. Notably, Ce^3+^ doping not only effectively quenched the persistent luminescence but also maintained the intrinsic radioluminescence efficiency of the Tb^3+^ centers. Subsequently, the optimized nanocrystals were incorporated into a poly(vinyl alcohol) (PVA) matrix to fabricate transparent scintillator films with high nanoparticle-loading capacity. The resulting composite films exhibited negligible afterglow, high spatial resolution and excellent radiation stability, demonstrating significant potential for real-time X-ray imaging applications.

## 2. Materials and Methods

### 2.1. Materials

All reagents were used as received without any purification. Ammonium fluoride (NH_4_F, 90%), cerium Chloride (CeCl_3_·6H_2_O, 99.99%), yttrium chloride (YCl_3_·6H_2_O, 99.99%), terbium chloride (TbCl_3_·6H_2_O, 99.99%), lutetium chloride (LuCl_3_·6H_2_O, 99.99%) sodium oleate (C_18_H_31_O_2_Na, 98%), 1-octadecene (C_18_H_36_, ≥90%), glycerol (C_3_H_8_O_3_, 99%) were purchased from Shanghai Macklin Biochemical Co., Ltd., Shanghai, China. Polyvinyl alcohol (PVA-1788L) was purchased from Anhui Wanwei Group Co., Ltd., Chaohu, China. Cyclohexane (C_6_H_12_, AR) were purchased from Tianjin Fuyu Fine Chemical Co., Ltd., Tianjin, China. The oleic acid (OA, 90%) was purchased from Sigma-Aldrich Co., Ltd., St. Louis, MO, USA. Hydrochloric acid (HCl, 12 M in water) was purchased from Yantai Far East Fine Chemical Co., Ltd., Yantai, China.

### 2.2. Preparation of Ln^3+^-Doped Naluf_4_ Nanoparticles

The Ln^3+^-doped NaLuF_4_ nanoparticles were synthesized via a modified solvothermal method. First, 6.4 mmol of rare-earth chlorides (Lu_0.85-x_Tb_0.15_Ce_x_Cl_3_), 25.6 mmol sodium oleate, 20 mL of oleic acid, and 20 mL of 1-octadecene were mixed in a 100 mL flask. The mixture was heated to 150 °C for 1 h under vacuum with stirring. After cooling, a transparent lanthanide-oleate precursor was obtained. Then, 38.4 mmol of ammonium fluoride powders were added under N_2_, heated at 160 °C for 1 h, and degassed for 10 min. Next, the reaction mixture was heated to 320 °C for 30 min under N_2_ with stirring. After cooling, the nanocrystals were precipitated with ethanol, collected by centrifugation, washed with cyclohexane and ethanol three times, and finally dispersed in cyclohexane.

Subsequently, core–shell nanoparticles were synthesized using the obtained nanocrystals as seeds. Similarly, a transparent lanthanide-oleate precursor was prepared by reacting yttrium chloride with sodium oleate. The core nanocrystals were then introduced into this precursor solution. The mixture was heated to 80 °C for 10 min to evaporate the cyclohexane solvent. Next, 12.8 mmol of ammonium fluoride was added under a nitrogen atmosphere, and the reaction mixture was heated at 160 °C for 1 h, followed by degassing under vacuum for 10 min. Subsequently, under a continuous nitrogen flow, the temperature was raised to 290 °C and maintained for 120 min with vigorous stirring, after which the system was cooled to room temperature. Finally, the resulting nanocrystals were precipitated by adding ethanol, collected via centrifugation, washed three times with cyclohexane and ethanol to remove impurities, and finally re-dispersed in cyclohexane for storage or further use.

### 2.3. Preparation of Scintillator Screen

The scintillator screen was fabricated via a drop-casting method. In a typical procedure, ligand-free nanocrystals were uniformly dispersed in a PVA aqueous solution (100 mg/mL) at a mass ratio of 4:1 (NPs:PVA). The resulting homogeneous suspension was then transferred into a disposable polystyrene Petri dish. The composite film was then dried under ambient laboratory conditions for 3 h to obtain a transparent scintillator screen.

### 2.4. Characterization

Steady-state photoluminescence (PL) and radioluminescence (RL) spectra were recorded with a fluorescence spectrometer (FS5, Edinburgh Instruments, Livingston, UK) equipped with a UV light and portable X-ray tube (MAGPRO, Moxtek, Orem, UT, USA) as the excitation source, respectively. Transmission electron microscopy (TEM) images were acquired with a JEM-2100Plus microscope (JEOL, Tokyo, Japan) operated at 200 kV. Powder X-ray diffraction (XRD) patterns were collected at room temperature with an X-ray diffractometer (Rigaku Ultima IV, Tokyo, Japan) using Cu Kα radiation (λ = 1.5406 Å). The scanning rate was 10° min^−1^ with a step size of 0.02°. Optical photographs of the films were taken with a digital camera (Canon EOS 90D, Tokyo, Japan). X-ray imaging was carried out on digital camera (Canon EOS 90D) coupled with a portable X-ray tube (MAGPRO, Moxtek). The X-ray tube was operated at 50 kV and 100 μA.

## 3. Results and Discussion

**Figure 1:**
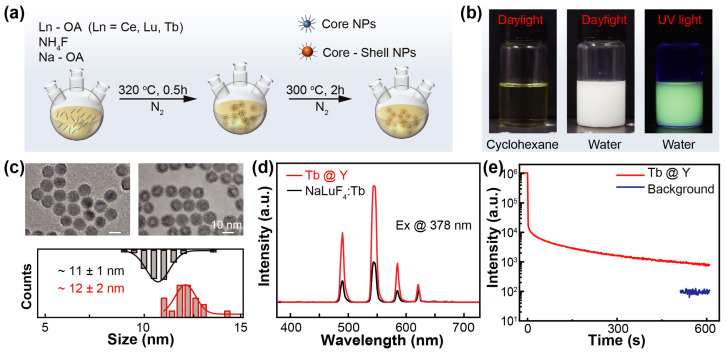
(**a**) Schematic diagram of the nanoparticle preparation process. (**b**) Luminescence images of the nanoparticles under sunlight and UV excitation. (**c**) TEM images and particle-size distributions of NaLuF_4_:Tb and NaLuF_4_:Tb@NaYF_4_ nanocrystals. (**d**) The luminescence spectra of NaLuF_4_:Tb and NaLuF_4_:Tb@NaYF_4_ nanocrystals. (**e**) The afterglow decay of NaLuF_4_:Tb@NaYF_4_ nanocrystals following 5 min of X-ray illumination.

To prepare Tb^3+^-doped NaLuF_4_ nanoparticles on a large scale, an improved solvothermal method was employed, as shown in Figure 1a. The optimal doping ratio of Tb^3+^ in NaLuF_4_ nanoparticles was determined to be 15% [26]. Exceeding this ratio reduces the luminescence intensity owing to self-quenching from interactions between Tb^3+^ ions. The synthesized nanoparticles were dispersed in cyclohexane to form a transparent and yellowish liquid. The nanoparticles were then modified with hydrochloric acid (2M), yielding a well-dispersed aqueous system that exhibited bright green emission under UV light (Figure 1b).

To enhance the luminescent performance, a core–shell architecture was fabricated by coating the nanoparticles with an inert NaYF_4_ layer. The resulting core–shell NaLuF_4_:Tb^3+^@NaYF_4_ nanoparticles had an average size of 12 nm, compared with 11 nm for the uncoated NaLuF_4_:Tb^3+^ cores (Figure 1c). The shell significantly increased the emission intensity by suppressing nonradiative recombination at surface defects (Figure 1d) [33,34]. Under X-ray excitation, the core–shell nanoparticles exhibited a strong afterglow lasting up to 600 s, with an intensity more than tenfold above the background signal (Figure 1e). However, this prolonged afterglow can degrade temporal resolution in dynamic X-ray imaging, underscoring the need for effective afterglow-suppression strategies to preserve image quality.

**Figure 2:**
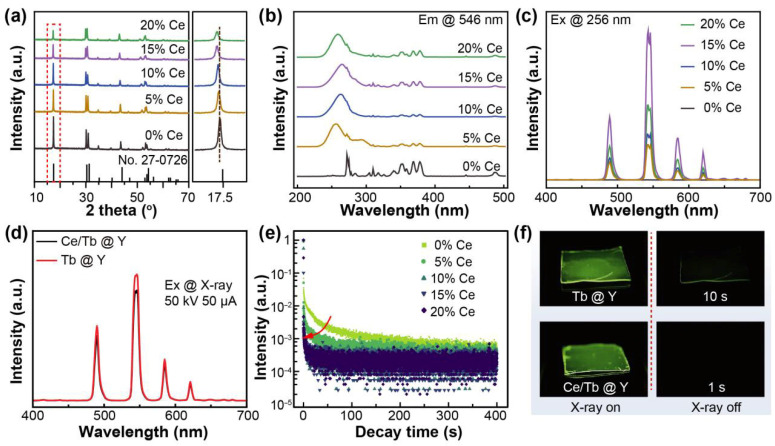
(**a**) X-ray diffraction (XRD) patterns, (**b**) photoluminescence excitation (PLE) spectra, and (**c**) photoluminescence (PL) spectra of a series of Ce-doped samples with varying doping concentrations (0%, 5%, 10%, 15%, and 20%). (**d**) The radioluminescence (RL) spectra of undoped and Ce^3+^-doped NaLuF_4_:Tb^3+^ nanoparticles. (**e**) The normalized afterglow of the series of Ce^3+^-doped samples with different doping concentrations. (**f**) The RL and afterglow images of the undoped and Ce^3+^ doped NaLuF_4_:Tb^3+^@NaYF_4_ nanoparticles.

To mitigate the afterglow, a series of Ce^3+^-doped NaLuF_4_:Tb^3+^@NaYF_4_ samples were synthesized. X-ray diffraction (XRD) patterns confirmed the phase purity of all samples, which matched the standard hexagonal NaLuF_4_ structure (JCPDS No. 27-0726) without detectable impurities (Figure 2a). As the Ce^3+^ concentration increased, the diffraction peaks shifted slightly to lower angles. This shift is attributed to lattice expansion caused by the substitution of smaller Lu^3+^ (0.86 Å) or Tb^3+^ (0.92 Å) ions with larger Ce^3+^ ions (1.01 Å). Photoluminescence excitation (PLE) spectra monitored at 546 nm showed characteristic Tb^3+^ excitation peaks, along with an emerging broad band ascribed to Ce^3+^ (Figure 2b). The presence of this Ce^3+^ band suggests potential energy transfer from Ce^3+^ to Tb^3+^, which is consistent with previous studies [35,36]. Under 256 nm excitation, the Tb^3+^ emission intensity increased with Ce^3+^ concentration and peaked at 15% doping (Figure 2c). Upon X-ray excitation, both doped and undoped samples exhibited similar emission profiles (Figure 2d). Notably, even at 15% Ce^3+^ doping, the RL intensity decreased only marginally, indicating that Ce^3+^ doping had no obviously effect on the scintillation efficiency of the Tb^3+^ ions.

As shown in Figure 2e, the afterglow decay results suggested that the incorporation of Ce^3+^ ions efficiently suppressed the long-lasting afterglow of NaLuF_4_:Tb^3+^@NaYF_4_ nanoparticles. As the Ce^3+^ concentration increased from 0 to 15%, the decay time (to 1% of the RL intensity) was decreased from 6 s to 0.1 s, corresponding to a 60-fold reduction. As the Ce^3+^ ions concentration exceeded 15%, the time decreased slightly while the RL intensity obviously decreased. Consequently, 15% Ce^3+^-doped NaLuF_4_:Tb^3+^@NaYF_4_ was chosen for the subsequent fabrication of the scintillator screen. Additionally, the RL and afterglow images of both the undoped and 15% Ce^3+^-doped NaLuF_4_:Tb^3+^@NaYF_4_ nanoparticles films showed a similar afterglow suppression (Figure 2f).

**Figure 3:**
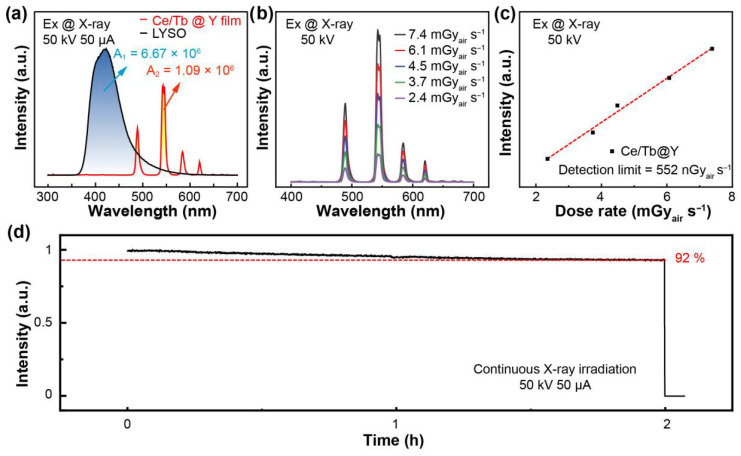
(**a**) Comparison of RL between a Ce^3+^-doped NaLuF_4_:Tb^3+^@NaYF_4_/PVA film (50 μm) and LYSO:Ce single crystal (500 μm). The integrated intensity (A1: 6.67 × 10^6^ and A2: 1.08 × 10^6^) was comparable despite a tenfold difference in thickness (50 μm vs. 500 μm). (**b**) RL of the Ce^3+^-doped NaLuF_4_:Tb^3+^@NaYF_4_/PVA film at various dose rates. (**c**) Detection limit of the Ce^3+^-doped NaLuF_4_:Tb^3+^@NaYF_4_/PVA film. (**d**) Radiation stability of the Ce^3+^-doped NaLuF_4_:Tb^3+^@NaYF_4_/PVA film under 50 keV and 50 μA.

Owing to the suppression of afterglow, Ce^3+^-doped NaLuF_4_:Tb^3+^@NaYF_4_ nanoparticles are a promising candidate for X-ray imaging. Typically, transparent nanocomposite films were fabricated by combining Ce^3+^-doped NaLuF_4_:Tb^3+^@NaYF_4_ nanoparticles with PVA using a simple drop-casting method. Compared with a commercial scintillator (i.e., 500 μm LYSO single crystal), the luminescence intensity of the film (50 μm) was comparable despite their large difference in thickness (Figure 3a). The dose-dependent RL of the Ce^3+^-doped NaLuF_4_:Tb^3+^@NaYF_4_/PVA film showed a linear response to the X-ray dose rate, as illustrated in Figure 3b,c. The calculated detection limit was 552 nGy_air_ s^−1^, approximately an order of magnitude lower than that required for medical diagnosis (~5.5 μGy/s) [37]. To assess its stability under X-ray irradiation, extended exposure tests were performed. After 2 h X-ray irradiation, the film maintains 92% of its initial luminescence intensity (Figure 3d).

**Figure 4:**
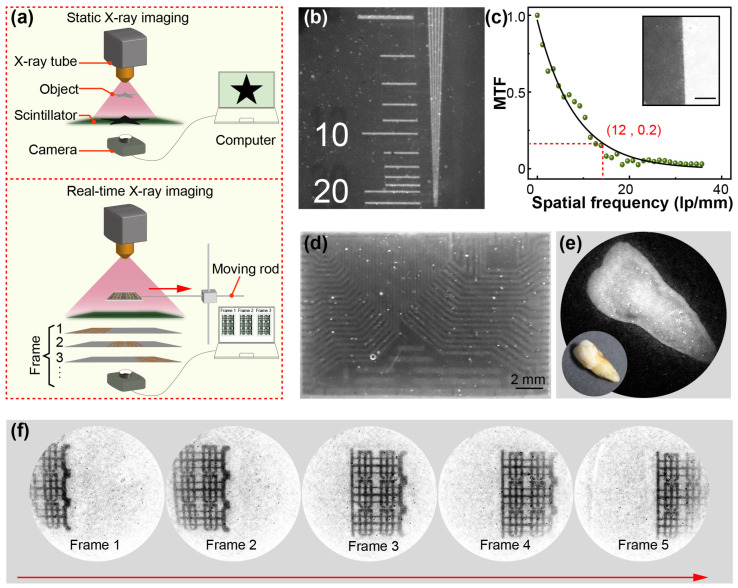
(**a**) Schematic diagram of static X-ray imaging and real-time X-ray imaging process. (**b**) Image of a resolution test target and (**c**) Modulation transfer functions (MTF) of a slanted-edge image revealed a spatial resolution of 12 lp/mm. (**d**) Static imaging line pattern of a line-pair pattern and (**e**) a circuit board. (**f**) Real-time X-ray imaging of a copper pattern on a moving rod, with images acquired at 1 s interval. (ISO 25600, F/2.8, exposure 1/60 s).

To further verify its X-ray imaging capabilities, a home-made X-ray imaging testbed was developed, consisting of an X-ray source, a Ce^3+^-doped NaLuF_4_:Tb^3+^@NaYF_4_/PVA film, and a digital camera (Figure 4a). In static X-ray imaging, the high transparency of the film enables the acquisition of high-quality images due to the reduced light scattering. To this end, resolution test targets and MTF were employed to evaluate the spatial resolution of the Ce^3+^-doped NaLuF_4_:Tb^3+^@NaYF_4_/PVA film, which exceeded 12 lp mm^−1^ (Figure 4b,c). Furthermore, an integrated circuit was employed in X-ray imaging, which demonstrated high resolution with distinct lines (Figure 4d). For biological-tissue imaging, an artificial-dentures sample was used, yielding similarly clear imaging results (Figure 4e).

To demonstrate real-time X-ray imaging capability, a moving rod was incorporated into a custom testbed designed for the Ce^3+^-doped NaLuF_4_:Tb^3+^@NaYF_4_/PVA scintillator screen. In a proof-of-concept experiment, a copper pattern was used as the imaging target, moving at a speed of 1 cm/s (Figure 4a). In the continuous-shooting experiment, the captured images showed no blurring or artifacts (Figure 4f and Video S1), indicating excellent real-time imaging performance even for moving targets. This fast temporal response was crucial for real-time X-ray imaging, as it enables the rapid conversion of X-rays to visible light and captures dynamic changes in the target. Overall, these results demonstrated outstanding real-time imaging capabilities, laying a solid foundation for further development and application in medical and industrial non-destructive X-ray imaging.

## 4. Conclusions

In summary, this work demonstrates an effective strategy for suppressing X-ray-induced afterglow in Tb^3+^-doped NaLuF_4_ nanocrystals via Ce^3+^ co-doping. The Ce^3+^ ions enabled efficient energy transfer and suppressed afterglow while largely preserving the radioluminescence intensity. When composited with PVA, the fabricated scintillator film exhibited a low detection limit of 552 nGy_air_s^−1^ and outstanding radiation stability, retaining 92% of its initial luminescence after prolonged X-ray irradiation. Static and dynamic imaging tests confirmed the high imaging capability of the material. Notably, a spatial resolution exceeding 12 lp/mm was achieved with negligible afterglow interference in real-time imaging. These results provide a viable route to developing high-performance scintillators for fast, high-resolution X-ray imaging applications such as medical diagnostics and non-destructive testing.

## Data Availability

The original contributions presented in this study are included in the article/Supplementary Materials. Further inquiries can be directed to the corresponding author.

## Supplementary Materials

The following supporting information can be downloaded at https://www.mdpi.com/article/10.3390/ma19020437/s1, Video S1: Low afterglow composite scintillator for real-time X-ray imaging.
